# Traumatic Anteromedial Radial Head Dislocation in an Adult: A Case of Brachialis Tendon Entrapment

**DOI:** 10.7759/cureus.3924

**Published:** 2019-01-21

**Authors:** Fırat Ozan, Kürşat Tuğrul Okur, Muhammed Melez, Ömer Can Ünlü, Kamil Yamak

**Affiliations:** 1 Orthopedics and Traumatology, Kayseri City Hospital, Kayseri, TUR; 2 Orthopedics and Traumatology, İzmir Bozyaka Training and Research Hospital, Izmir, TUR

**Keywords:** radial head dislocation, brachialis tendon interposition, irreducible joint dislocation, elbow, heterotopic ossification

## Abstract

Isolated traumatic anteromedial radial head dislocation is an uncommon injury in adults. The brachialis tendon interposition rarely interferes with the radial head reduction procedure. In the present paper, we report the case of an 18-year-old male who sustained an injury to his right elbow during a wrestling match and developed isolated anteromedial radial head dislocation. Open reduction had to be performed due to entrapment of the radial head at the brachialis tendon.

## Introduction

Traumatic radial head dislocation is usually observed with Monteggia type fractures, and children are more likely to suffer with isolated radial head dislocation than adults [1-3]. Isolated radial head dislocation without fracture and bowing in the ulnar bone is a rare occurrence in adults, with posterior dislocation being a more common manifestation [1-7].

Isolated traumatic anteromedial radial head dislocation in adults is rarely reported in the literature [4-5]. Only two cases of an irreducible anteromedial radial head dislocation due to soft tissue interposition have been reported in adult patients [4-5]. Moreover, inability to perform the radial head reduction due to brachialis tendon interposition is a very rare situation, with only one such case previously published in the literature [1, 4-7].

In this study, we report a rare case of isolated anteromedial radial head dislocation in an adult. Open reduction was performed due to entrapment of the radial head at the brachialis tendon.

## Case presentation

An 18-year-old male patient presented to our emergency polyclinic with pain, swelling, deformity, and limited joint mobility in the right elbow. He had sustained an injury to his right elbow during a wrestling match. He had fallen backwards on an outstretched hand with his wrist in dorsiflexion and hyperpronation. The patient had a restricted active range of motion, especially the supination-pronation movements of the forearm. However, flexion-extension movements of the elbow joint were intact. Ecchymosis was present on the anteromedial aspect of the right elbow. Neurovascular status of the limb was normal.

**Figure 1:**
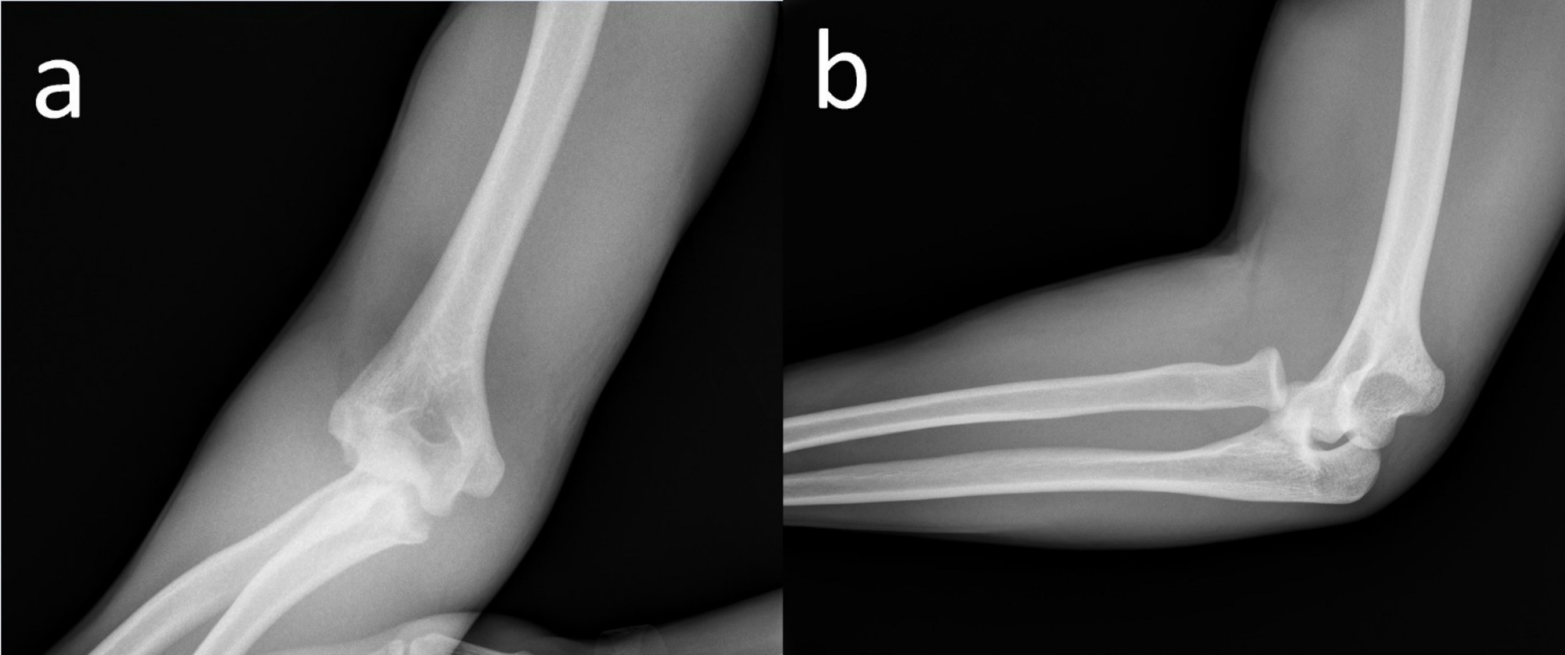
Radiographic images of the patient immediately after injury. Anteroposterior (a) and lateral (b) radiographic images of the patient indicating isolated anteromedial radial head dislocation of the right elbow.

X-ray images indicated isolated anteromedial radial head dislocation (Figure 1).

**Figure 2:**
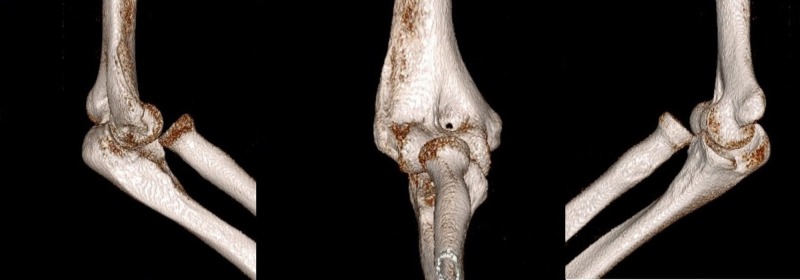
Computed tomography (CT) images of the elbow show isolated traumatic anteromedial radial head dislocation.

A computed tomography (CT) scan of the elbow was performed (Figure 2).

Closed reduction was attempted in the emergency room using various maneuvers; however, successful reduction could not be achieved.

**Figure 3:**
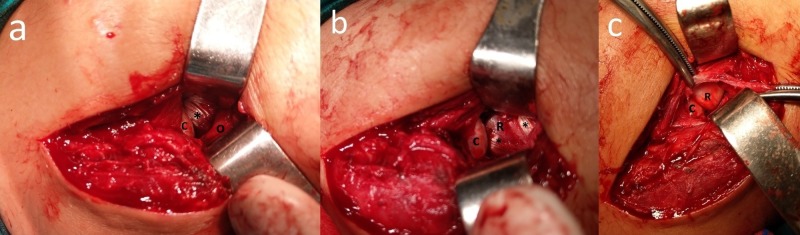
Intraoperative view of the patient. (a, b) demonstrating the brachialis tendon wrapped around the radial head (C: *Capitellum*, R: Radial head, ∗: Brachialis tendon, O: Olecranon); (c) Appearance of the radiocapitellar joint after reduction.

Thus, an open reduction was considered. Boyd’s approach was used to expose the radial head. A plane was made between the extensor carpi ulnaris and anconeus, and the radiocapitellar joint was exposed. We found that the brachialis tendon was wrapped around the radial neck and noted that the tendon pulled the dislocated radial head anteromedially. The brachialis tendon restricted radial head reduction. We also detected that the annular ligament was ruptured. The brachialis tendon was released from the radial head and the joint was reduced (Figure 3).

**Figure 4:**
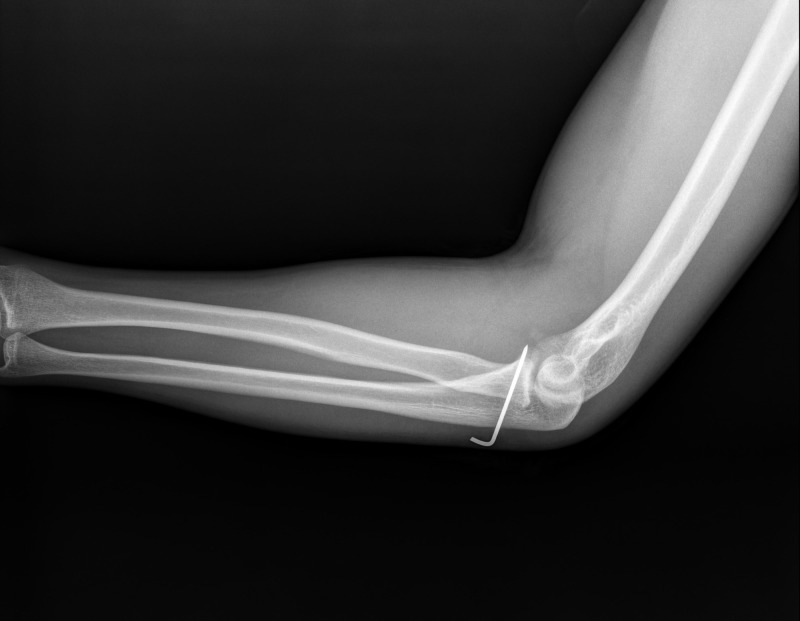
Postoperative radiographic images of the patient. Radiographic image of the patient shows a K-wire was used to maintain the stability of the reduction of the proximal radioulnar joint.

However, the reduction was unstable. Therefore, the annular ligament was repaired and a radioulnar Kirschner wire (K-wire) was used to maintain reduction of the proximal radioulnar joint (Figure 4).

**Figure 5:**
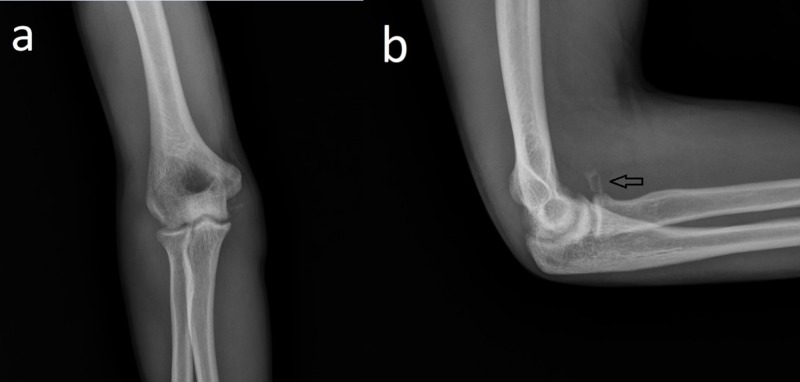
Radiographic images at follow-up. Radial head in reduced position (a, b). Minimal ectopic ossification developed at the anterior of the joint capsule (arrow).

Postoperatively, a hinged long arm cast brace was applied and the patient was allowed to perform flexion-extension movements. Ectopic ossification was observed anterior to the joint at the time of first follow-up. Then, a single dose of 7-Gy radiotherapy was administered to the patient. No progression was detected in ectopic ossification at the subsequent follow-up. The wire and cast brace were removed after four weeks and elbow mobilization was initiated (Figure 5).

**Figure 6:**
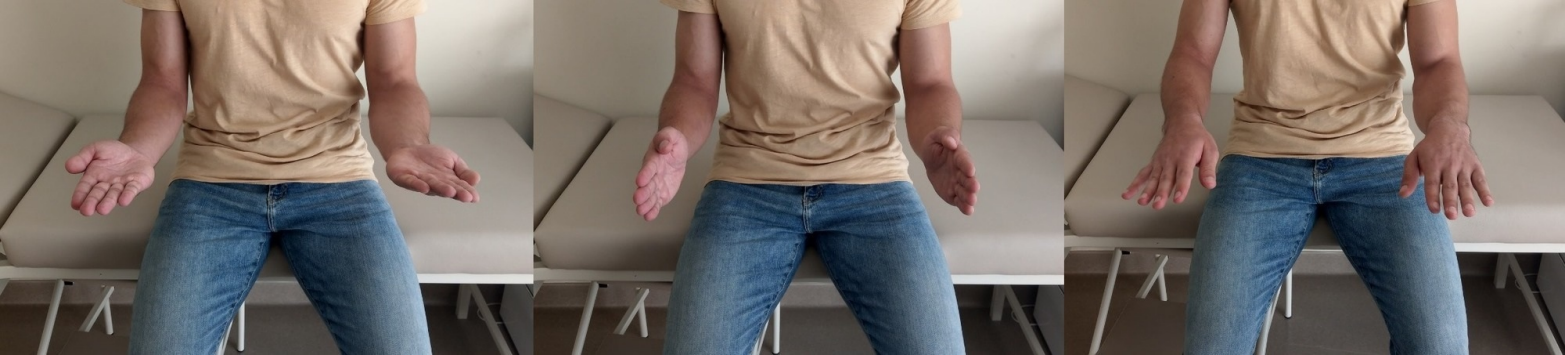
Postoperative upper-extremity images. The appearance of the patient’s right elbow range of motion at the end of follow-up.

During the course of subsequent follow-ups, it was observed that the patient had gradually resumed his normal activities. The functional outcome of the patient improved without pain or disability (Figure 6).

## Discussion

Traumatic radial head dislocation is usually associated with fractures of the forearm [1, 4-8]. Isolated radial head dislocation in adults is rare [1-8]. Only a few cases of truly isolated traumatic anteromedial or anterior radial head dislocation have been reported (Table 1) [1, 4-7]. 

**Table 1:** Studies available in the literature on irreducible isolated traumatic anteromedial or anterior radial head dislocation due to soft tissue interposition reported in adult patients.

Study (year)	Sex	Mean age (years)	Type of dislocation	Type of soft tissue interposition	Treatment
Kansay et al. (2018) [6]	Male	18	Anterior dislocation	Anterior joint capsule	Open reduction - repair of the annular ligament - Kirschner wire fixation
Climent-Peris et al. (2016) [4]	Female	14	Anteromedial dislocation	Biceps tendon	Open reduction - repair of the annular ligament
Cates et al. (2016) [5]	Male	16	Anteromedial dislocation	Brachialis tendon	Open reduction
Watanabe et al. (2005) [1]	Male	24	Anterior dislocation	Annular ligament	Open reduction - repair of the annular ligament
Takami et al. (1997) [7]	Male	20	Anterior dislocation	Annular ligament	Open reduction - Kirschner wire fixation

Closed reduction has been shown to be successful in most isolated radial head dislocation cases [4-6]. Inability to perform the reduction procedure due to brachialis tendon interposition is a rare situation, with only one previously published case in the literature [5]. Interposition of the biceps tendon, annular ligament, and anterior capsule have also been described as causes of irreducible anterior radial head dislocation [1-4, 6-7].

Radial head dislocations can usually be reduced by simple maneuvers [1, 4-6]. However, surgery may be necessary to reduce radial head dislocations transposed with soft tissue [6]. In very few cases, open reduction has been required [1-7].

Radial head dislocations are caused by a variety of mechanisms [1, 3-7, 9-10]. Injury resulting from a fall in a hyperextended and supination position may cause anterolateral radial head dislocation [3-4, 6, 9]. This situation may be followed by hyperpronation which displaces the head medially, and subsequent ﬂexion of the elbow entraps the tendon around the radial neck [3-4, 6, 9]. This blocks the head in a medial position and prevents closed joint reduction [3-4, 6, 9]. Takami et al. reported a case of a patient with an isolated anterior radial head dislocation after a direct blow to the posterior aspect of a semi-flexed elbow [7]. Salama et al. reported an anterior radial head dislocation after a biceps contraction caused by an electric shock [10]. Sasaki et al. suggest that hyperpronation plays an important role in causing such an injury [3]. In our case, the mechanism of dislocation was considered to be related to extension of the elbow and dorsiflexion-hyperpronation of the wrist.

The annular ligament is the primary stabilizer of the proximal radioulnar joint [1, 7]. Other structures such as the quadrate ligament and interosseous membrane also provide stability to this joint [9]. The annular ligament provides multidirectional stability to the proximal radioulnar joint in patients with gross instability of the radial head [9]. Thus, the authors recommend performing annular ligament repair [6]. Radial head dislocation is usually accompanied by damage to the annular ligament. In the presented case, the annular ligament was found to be disrupted and was, therefore, repaired.

Radial head instability can sometimes develop after reduction [1-7, 11]. Therefore, treatments such as radial head excision, K-wire fixation, and modified Bell-Tawse repair have been proposed [1-7, 11]. In the presented case, after repairing the annular ligament, K-wire fixation was performed for three weeks to create a stable reduction.

Ectopic ossification can often appear after joint dislocation [1]. Accordingly, in our case, an ectopic ossification appeared at the time of follow-up. A single dose of 7-Gy radiotherapy was administered to the patient. No progression in ectopic ossification was detected at subsequent follow-up.

## Conclusions

We presented a rare case of isolated radial head dislocation in an adult patient. The brachialis tendon was wrapped around the radial head, pulling the dislocated radial head anteromedially. We suggest that transposed brachialis tendon or other soft tissues should be taken into consideration in the management of a radial head dislocation if radiographs indicate that the radial head is dislocated anteromedially. Consequently, surgical reduction may be required in such cases.
